# Water Parameters Predicting the Seasonal and Spatial Dynamics of the Vibrio Harveyi- and Splendidus-Clade Pathogens

**DOI:** 10.3390/microorganisms13092167

**Published:** 2025-09-17

**Authors:** Karagan Royer, Andrei L. Barkovskii

**Affiliations:** Department of Biological and Environmental Sciences, Georgia College and State University, Milledgeville, GA 30161, USA

**Keywords:** *V. parahaemolyticus*, *V. alginolyticus*, *V. harveyi*/*V. campbellii*, *V. splendidus*, distribution, virulence, prediction, water, sediment, oysters, clams

## Abstract

*Vibrio* species of the Harveyi and Splendidus clades are the causative agents of vibriosis, resulting in mortality rates of up to 100% in common aquaculture species. They are primarily responsible for seafood-related illnesses in humans, causing gastroenteritis. Except for *V. parahaemolyticus*, the ecological behaviour of these pathogens is poorly understood. We investigated the spatial and temporal distribution of *V. parahaemolyticus*, *V. alginolyticus*, *V. harveyi*/*V. campbellii,* and *V. splendidus* in three Georgia (USA) grounds for *Crassostrea virginica* and *Mercenaria mercenaria*. DNA from oysters, clams, water, and sediment was collected over a year-long study and analyzed using quantitative PCR (qPCR) to assess the prevalence and concentrations of the above *Vibrio* species. The study targeted the *tlh*, *VA1198230*, *rpoA*, and *recA* genes using species-specific primers. Species abundance was estimated based on the concentrations of the corresponding genes. The species abundance was profiled for water parameters and concentrations of the clade-specific virulence genes *toxR*, *luxR*, *srp*, *vhhA*, *vhh*, and *vhp* that were previously detected in the study area. *V. parahaemolyticus* was the most common species, detected year-round in 61% and 44% of the water and sediment samples, respectively, followed by *V. splendidus* (67% and 17%) and *V. harveyi*/*V. campbellii* (19% and 33%). *V. alginolyticus* was rarely detected in water and never in sediment. In bivalves, the highest frequency was observed for *V. parahaemolyticus*. This species was detected in 89% of clam and 100% of oyster samples, followed by *V. alginolyticus* (22% and 17%) and *V. splendidus* at 17% in both species. No *V. harveyi*/*V. campbellii* has been detected in clams and oysters. Seasonal dynamics and concentrations varied between the species. Water temperature (r = 0.58–0.63, *p* ≤ 0.05), pH (r = −0.46), and dissolved oxygen (r = −0.42 to −0.56, *p* ≤ 0.05) were reliable predictors for the abundance of the Harveyi and Splendidus clade pathogens in bivalves and the water column, but not in sediments. In water and sediments, the abundances of *V. harveyi*/*V. campbellii* and *V. parahaemolyticus* were highly correlated (r = 0.80–0.99, *p* ≤ 0.001) to concentrations of most of the virulence genes, with some heterogeneities between the sites. The study revealed the species-specific dynamic of the Harveyi and Splendidus clade pathogens, provided the first evidence for the presence of *V. harveyi*/*V. campbellii* in the Atlantic USA waters, and identified environmental predictors for monitoring the Harveyi and Splendidus clade pathogens in mollusks and the water column.

## 1. Introduction

The Harveyi clade is the second-largest clade of the *Vibrio* genus and is composed of 12 non-pathogenic and pathogenic species [1]. The latter share a signature array of virulence genes and cause infections and diseases in fish and shellfish [2], as well as human gastroenteritis, diarrhea, nausea, and vomiting due to the consumption of raw and undercooked seafood [3,4].

In a previous study [5], numerous clade-specific virulence genes were detected in the water and sediment of three Georgia (USA) clams and oyster grounds. These genes were detected with Harveyi-clade-specific primers [6], and their patterns revealed the diversification and possible stratification of their carriers into different ecological niches in response to changes in water parameters, such as temperature, pH, salinity, and turbidity [5]. The most common Harveyi-clade species associated with shellfish and human diseases are *Vibrio harveyi*, *Vibrio alginolyticus*, *Vibrio campbellii*, and *Vibrio parahaemolyticus* [2].

*Vibrio parahaemolyticus* is one of the best-known species in this clade. This is primarily associated with its role in infecting humans through the consumption of raw or undercooked seafood [7]. Gastroenteritis is a common symptom of *V. parahaemolyticus* infection, but immunocompromised patients can exhibit more severe symptoms such as septicemia and multi-organ failure [8]. The symptoms vary depending on the route of transmission. *V. parahaemolyticus* is also associated with wound infections, where individuals encounter contaminated water while having a cut or lesion on their skin [9]. Although uncommon, *V. parahaemolyticus* can cause more severe conditions, such as soft tissue damage and necrotizing fasciitis [10,11]. Of the 80,000 cases of vibriosis in the USA, 40% are caused by *V. parahaemolyticus* [12].

*Vibrio alginolyticus* is the causal agent of shellfish and fish diseases [13]. This pathogen is a major contributor to mass mortality in *Crassostrea gigas* aquaculture [14,15] and fish species [16]. *V. alginolyticus* is pathogenic to humans and causes gastrointestinal and other infections [17]. Of the 80,000 cases of vibriosis in the USA annually, 20% are caused by *V. alginolyticus* [12]. Studies have shown a notable increase in the number of cases of otitis externa during warmer months, with *V. alginolyticus* being the causative agent [18]. Between 1998 and 2012, 30 percent of soft tissue and bloodstream infections caused by *V. alginolyticus* in the USA were reported along the Atlantic coast [17]. Surprisingly, there are no published studies on its distribution and abundance in the Atlantic coastal waters of the Southeast United States.

*Vibrio harveyi* and *Vibrio cambellii* are known to affect shrimp, fish, and mollusks in Southeast Asia [19,20,21], leading to significant economic losses. Most infected marine species exhibit lethargy, luminescence, and tissue necrosis, with an 80% mortality rate in shrimp [22], and cause rare incidences of vibriosis in humans [23]. These two *Vibrio* species, with more than 98% 16S rRNA gene similarity [20] form a tight subclade within the Harveyi clade [24], which complicates species-level differentiation. *V. harveyi* and *V. campbellii* also share the same virulence factors [19]. Because of its high similarity to *V. harveyi*, diseases caused by *V. campbellii* may be severely underreported [24,25]. Both species have not been previously reported in Atlantic USA waters, but their spread to these waters seems possible due to climate change-related expansion.

*V. splendidus* is part of the Splendidus clade, is commonly found in coastal regions, and mostly infects bivalves, such as oysters and clams [14,26]. Common hosts for this species include the Pacific oyster (*Crassostrea gigas*) and carpet shell clam (*Ruditapes decussatus*) [14,27], causing mass mortality in both clams and oysters and substantial economic losses in aquaculture. While known in the North Atlantic [28], there are no reports on its presence and abundance in the Southeastern USA Atlantic.

While *V. parahaemolyticus* has been repeatedly described in the coastal waters of the Southeastern USA, including the study area [29], the presence and distribution of other Harveyi-clade pathogenic species in this environment have not yet been documented. A previous study revealed the presence of a wide array of Harveyi-clade-specific virulence genes in the study area [5]. This observation justifies the study on the presence and distribution of the Harveyi-clade pathogenic species in Southeastern USA waters. In the current study, we aimed to evaluate the presence and temporal distribution of the major Harveyi-clade pathogens, *V. parahaemolyticus*, *V. alginolyticus*, and *V. harveyi*/*V. campbellii* in oysters, clams, water, and sediments of the coastal waters of Georgia (USA). *V. splendidus* was added to the study as a well-known pathogen of oysters and clams [14,26] that has not been previously evaluated in the Southeastern Atlantic waters of the USA. We also aimed to attribute the previously detected Harveyi-clade-specific virulence genes to their Harveyi-clade carriers and to identify water parameters predictive to the presence and abundance of the above pathogens in the water column, oysters, and clams.

## 2. Materials and Methods

### 2.1. Field Sites and Sampling Events

**Figure 1:**
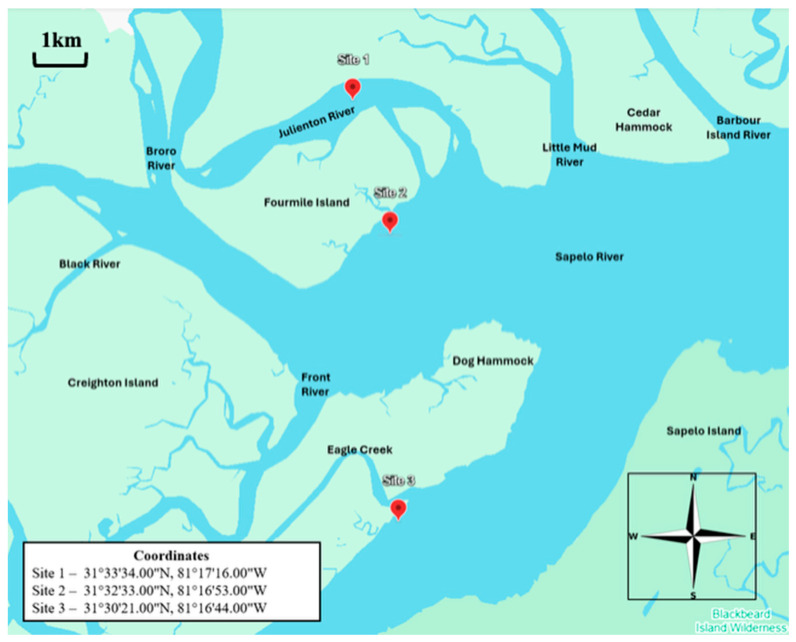
Study sites.

Three sampling sites located offshore of the coastal marshes of Townsend, Georgia, USA, were used to collect water, sediment, oyster (*Crassostrea virginica*), and clam (*Mercenaria mercenaria*) samples (Figure 1). Site 1 was located north of Fourmile Island along the Julienton River (31°33′34 N, 81°17′16 W). Site 2 was located at the mouth of the Sapelo River (31°32′33 N, 81°16′53 W). Site 3 was the southernmost site, with coordinates of 31°30′21 N, 81°16′44 W, located along the Mud River. All three sampling sites were tidal rivers that feed into Sapelo Sound Bay. Oysters and clams were collected from privately owned beds operated by Sapelo Sea Farms. Six sampling events occurred during low tide between alternating months from June 2022 to April 2023. DNA obtained from these samples has been previously evaluated on the abundance and divergence of the Haveyi-clade-specific virulence genes [5] in the above sites, preserved at −80 °C, and used for the speciation of Vibrio pathogens and their dynamics in the current study.

### 2.2. Water Collection, Processing, and Storage of Samples

Water samples were collected in triplicate from three segments of each site. Nine water samples were collected at each site in each sampling month. Before collection, sterile bottles were rinsed with environmental water. Approximate water depth at low tide was around 3 m. Water was collected in 1 L sterile glass bottles at a depth of ~1.5 m using a 4000 Subsurface Grab Bottle Sampler (Conbar, Monroeville, NJ, USA). Each triplicate was taken ~25 m apart to ensure an accurate representation of the site [30]. After collection, the samples were immediately placed on ice and, within 12 h, transported to the laboratory, where filtration and DNA extraction took place immediately after arrival. The samples were filtered through a custom water filtration system using 0.22 μm Millipore nitrocellulose filters (Millipore Sigma, Burlington, MA, USA). Using the Qiagen DNeasy^®^ PowerWater kit (Venlo, The Netherlands), total DNA was extracted then quantified using a NanoDrop ND-1000 Spectrophotometer (Thermo Fisher Scientific^®^, Waltham, MA, USA). The extracted DNA was stored at −20 °C.

### 2.3. Sediment Collection, Processing, and Storage of Samples

In each sampling event, sediments were collected at low tide in triplicate from three sections of each of the three sites. To account for site variation, each triplicate was taken from a different clam or oyster bed within ~25 m of each other [30]. Sediments were collected directly from the shallow segments of these beds at a depth of 10–20 cm. Sterile gloves and sterile double-ended laboratory spoons (Cole-Parmer, Vernon Hills, IL, USA) were used to retrieve the sediment samples and packed into sterile 1.5 mL microcentrifuge tubes. The tubes were placed on ice in Nasco Whirl-Pak bags (Chicago, IL, USA) for transportation to the laboratory. After reaching the laboratory, 0.25 g of sediment was used to extract DNA using the Qiagen DNEasy^®^ PowerSoil Kit (Venlo, The Netherlands), following the manufacturer’s protocol. Total DNA was quantified using a NanoDrop ND-1000 Spectrophotometer (Thermo Fisher Scientific, Waltham, MA, USA), and the samples were stored at −20 °C.

### 2.4. Oyster & Clam Collection, Processing, and Storage of Samples

Oysters and clams were collected manually from each site. Triplicates of five mollusks were harvested from random oyster and clam beds at each of three sites to cover the natural variation of the site. The oysters and clams were placed on ice in separate Nasco Whirl-Pak bags (Chicago, IL, USA) and transported to the laboratory. Once in the lab, the mollusks were aseptically shucked and drained. The entire visceral tissue of five mollusks per sample was collected and rinsed with a 0.8% NaCl solution. The visceral tissue was then diluted with an equal volume of sterile phosphate-buffered saline solution (PBS) to make a 1:1 (wt/vol) dilution (pH 7.4, Fisher Scientific). The tissues were homogenized for 1 min using a Tissue Tearor (Biospec Products, Bartlesville, OK, USA) at a mid-range speed setting. The homogenate (0.025 g) was used to extract DNA using the Qiagen DNeasy^®^ Blood & Tissue Kit (Venlo, The Netherlands). Total DNA was quantified using a NanoDrop ND-1000 Spectrophotometer (Thermo Fisher Scientific, Waltham, MA, USA) and stored at −20 °C.

### 2.5. Environmental Parameters

The environmental parameters for each site and each month (except April) were recorded using a Horiba-52G Multiparameter Water Quality Meter (Horiba Advanced Techno Co., Ltd., Kyoto, Japan) at an approximate depth of 1.5–2 m. Temperature (°C), pH, salinity (ppt), turbidity (NTU), dissolved oxygen (mg/L), total dissolved solids (g/L), conductivity (mS/cm), and potential water density (∂t) data were collected at each site Table S1).

### 2.6. Primers and PCR Protocols

Published species-specific primers for the Harveyi clade were selected after careful consideration and were retested for their validity in our lab. The reference species of *V. harveyi* (ATCC 14126), *V. campbellii* (ATCC BAA-1116/BB120), *V. splendidus* (ATCC 33125), *V. parahaemolyticus* (ATCC 17802), and *V. alginolyticus* (ATCC 17749) were used for retesting several primers against each species under the study. The species-specificity of the primers, the absence of cross-reactions with other species, and detection thresholds were examined. The reference cultures were grown in Difco Marine Broth 2216 (Franklin Lakes, NJ, USA) overnight, at optimal temperatures for each species. The pure cultures were subjected to DNA extraction using the Qiagen DNeasy Microbial Kit (Venlo, The Netherlands), followed by DNA quantification using a NanoDrop ND-1000 Spectrophotometer (Thermo Fisher Scientific^®^, Waltham, MA, USA). Extracted DNA concentration ranged from 20 to 100 ng/μL. An assay of 5 μL Bio-Rad PCR Master Mix (Hercules, CA, USA), 3 μL of nuclease-free water, 1 μL of each primer (200 nm), and 1 μL of DNA was assembled to perform PCR. DNA from *Escherichia coli* ATCC 11775™ was used as a negative control in all the test runs.

Several primers failed to differentiate between the reference species or had a low detection threshold. The primers used in this study (Table S2) and the cycling parameters for qPCR (Table S3) [6,31,32,33] are provided in the Supplementary Materials.

After purification with the Qiagen QIAquick PCR purification kit (Venlo, The Netherlands), the PCR products were quantified using a NanoDrop ND-1000 Spectrophotometer (Thermo Fisher Scientific^®^, Waltham, MA, USA). The formula used to calculate gene copy number was as follows: number of copies = (ng of DNA × (6.022 × 10^23^)/(size of amplicon in bp × (1.0 × 10^9^) × 650). The gene copy numbers in purified DNA ranged from 3.20 × 10^11^ to 1.62 × 10^12^ per ng. Each DNA sample was serially diluted and used to generate standard curves.

A qPCR protocol was developed for each target gene, and standard curves were generated. The dilutions were run in triplicate using dye-based qPCR. The assay included 1 μL of the targeted DNA, 5 μL of the Bio-Rad SYBR Green Master Mix (Hercules, CA, USA), 3.75 μL of nuclease-free molecular-grade water, 1 μL of BSA (0.5 mg/μL), 0.25 μL of MgCl_2_ (25 mM), and 0.2 μL of the corresponding primer set. A melt curve step was added to the cycling parameters to analyze the qPCR products.

The MASTERO Bio-Rad software 2.3 featured a linear regression of the log copy number values and the cycle threshold (ct) values for each assay was applied. The R^2^ value and y-intercept from the automated equation were used to calculate the gene copy numbers for the collected environmental samples.

The copy number per well was used as the base value to calculate the copy number per 1 mL of water and 1 g of sediment, clams, and oysters. The formula used to calculate copy number per well is as follows: copy number per well = 10^((Cq−y-intercept)/(-slope))^.

### 2.7. Statistical Treatment of Data

The graphs were generated using the RStudio software (R version 2024.12.0+). Pearson’s coefficient and *p*-values were used to evaluate the correlation between average copy numbers and water parameters. Similarly, Pearson’s statistic was used to correlate the abundance of the previously detected virulence genes and species-specific copy numbers in the study area, with *p*-values applied. The abundance of the *Vibrio* species was compared to the abundance of previously detected Harveyi-clade-specific virulence genes [5] in the same samples.

## 3. Results

### 3.1. Frequency of Detection of the Targeted Vibrio Species

All targeted *Vibrio* species were detected in all three sites. *V. parahaemolyticus* was the most common, detected year-round in 61% and 44% of the water and sediment samples, respectively, followed by *V. splendidus* (67% and 17%), and *V. harveyi*/*V. campbellii* (19% and 33%) (Table 1). *V. alginolyticus* was rarely detected in water and never in sediment. In bivalves, the highest frequency was observed for *V. parahaemolyticus*. This species was detected in 89% of clam and 100% of oyster samples, followed by *V. alginolyticus* (22% and 17%) and *V. splendidus* at 17% in both species (Table 2). No *V. harveyi*/*V. campbellii* has been detected in clams and oysters.

### 3.2. Seasonal and Spatial Dynamics of V. splendidus and the Harveyi-Clade Species

#### 3.2.1. *Vibrio splendidus*

**Figure 2:**
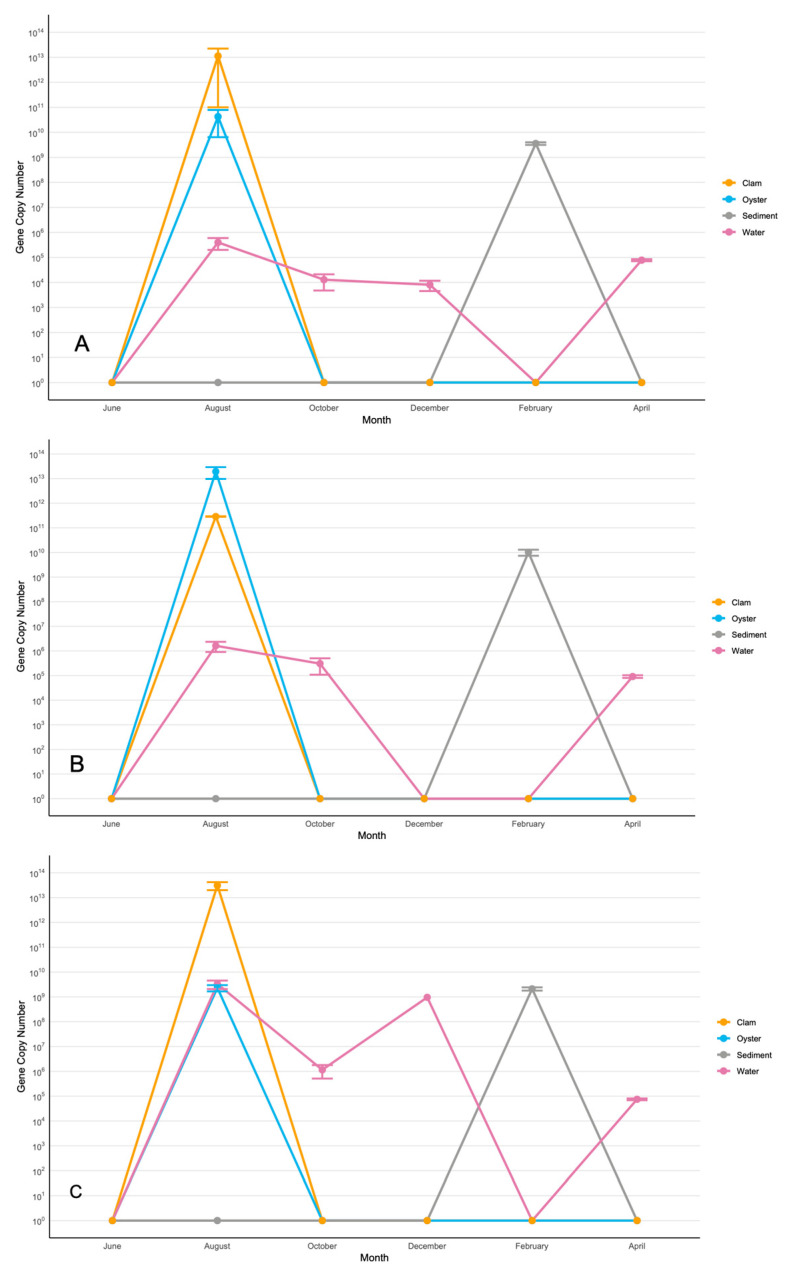
Copy numbers/g/mL of *V. splendidus* in Sites 1 (**A**), 2 (**B**), and 3 (**C**). The data for the figure were obtained from samples collected in triplicate from three segments of each site. Nine samples were collected at each site in each sampling month. Each triplicate was taken ~25 m apart to ensure an accurate representation of the site. Average copy numbers were displayed per 1 g of sediment, clams, and oysters and 1 mL of water. Error bars represent standard deviations (N = 9).

In all three sites, *V. splendidus* displayed remarkably similar spatial and temporal dynamics (Figure 2). *V. splendidus* was detected in the water column most months, with peaks in August and April. The August peak of its water concentration at 10^6^–10^9^ gene copies/mL coincided with its peak in bivalves at 10^11^–10^13^ gene copies/g. The second peak in April did not correspond to a spike of *V. splendidus* in the bivalves. In sediments, *V. splendidus* occurred only in February at 10^9^–10^10^ gene copies/g. No *V. splendidus* was detected in the bivalves or the water column in the winter months. These seasonal and spatial dynamics of *V. splendidus* suggest the water column as a source of this species to oysters and clams in summer months, and sediments as the main ecological niche for *V. splendidus* in winter.

#### 3.2.2. *Vibrio alginolyticus*

**Figure 3:**
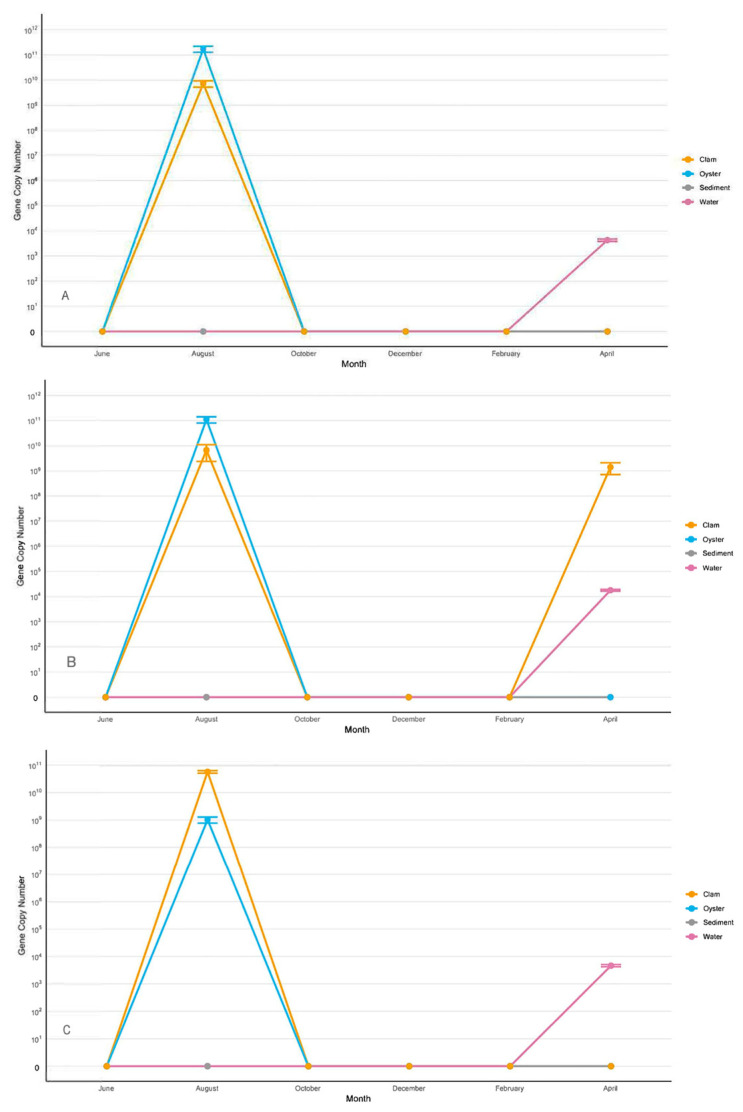
Copy numbers/g/mL of *V. alginolyticus* in Sites 1 (**A**), 2 (**B**), and 3 (**C**). The data for the figure were obtained from samples collected in triplicate from three segments of each site. Nine samples were collected at each site in each sampling month. Each triplicate was taken ~25 m apart to ensure an accurate representation of the site. Average copy numbers were displayed per 1 g of sediment, clams, and oysters and 1 mL of water. Error bars represent standard deviations (N = 9).

In all three sites, *V. alginolyticus* displayed remarkably similar spatial and temporal dynamics that were distinctive from that of *V. splendidus* (Figure 3). As for *V. splendidus,* this species was detected in oysters in clams in August at the overall highest concentration of 10^9^–10^11^ gene copies/g. In contrast to *V. splendidus,* no presence of *V. alginolyticus* at that time was revealed in the water column. In the water column, *V. alginolyticus* was detected at ~10^4^ gene copies/mL in April, which coincided with its spike in clams at ~10^9^ gene copies/g, but only in one site (Figure 3B). *V. alginolyticus* was the only *Vibrio* species not detected in sediments.

#### 3.2.3. *Vibrio harveyi/Vibrio campbellii*

**Figure 4:**
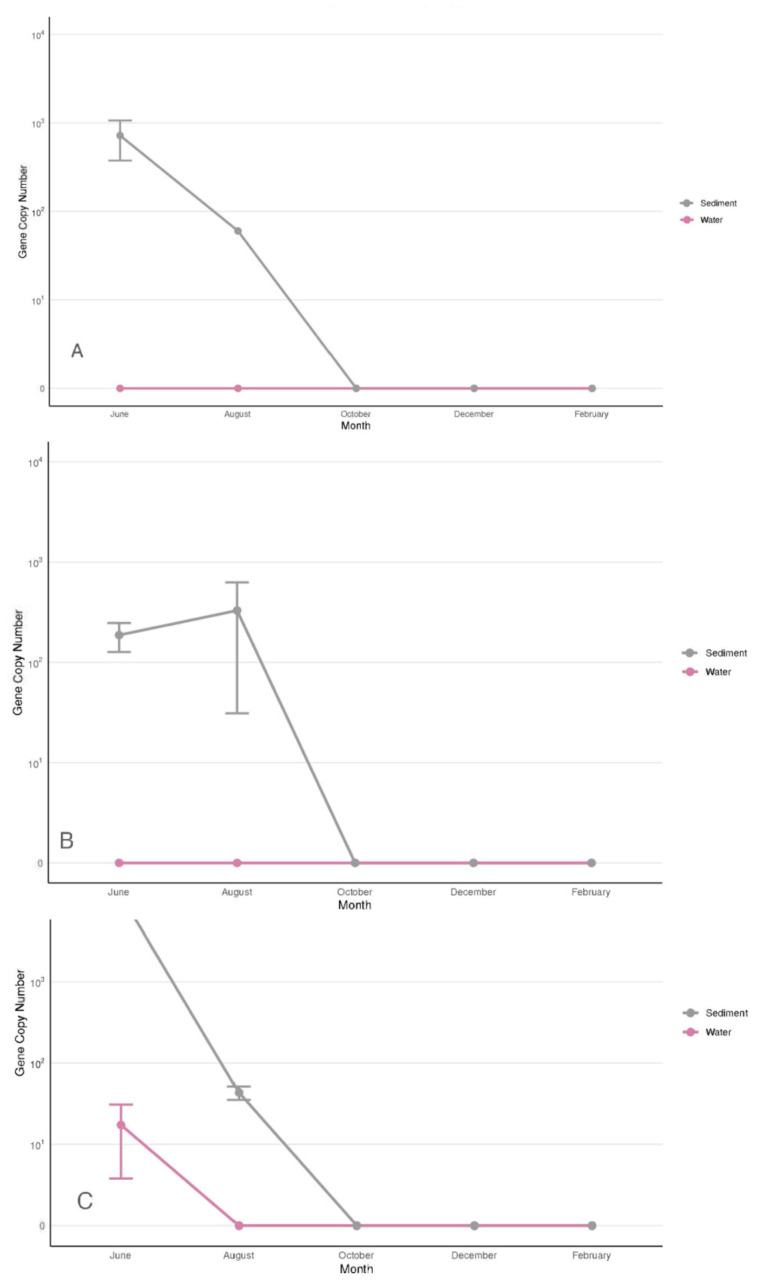
Copy numbers/g/mL of *V. harveyi*/*V. campbellii* in Sites 1 (**A**), 2 (**B**), and 3 (**C**). The data for the figure were obtained from samples collected in triplicate from three segments of each site. Nine samples were collected at each site in each sampling month. Each triplicate was taken ~25 m apart to ensure an accurate representation of the site. Average copy numbers were displayed per 1 g of sediment, clams, and oysters and 1 mL of water. Error bars represent standard deviations (N = 9).

The current study is the first report on the presence of *Vibrio harveyi/Vibrio campbellii* on the Atlantic coast of the USA. The trend exhibited by *V. harveyi*/*V. campbellii* differed from all the other *Vibrio* species in this study (Figure 4). These species were observed in all three sites but only between June and August. During that time, *V. harveyi*/*V. campbellii* resided mostly in sediments with gene copies of 10^3^–10^4^ per gram. June was the only month when these species were recorded in the water column in Site 3 (Figure 4C). Within the site, its concentration was low and varied between the samples in the range of 5–50 gene copies/mL (Figure 4C). No *V. harveyi*/*V. campbellii* have been detected in oysters and clams.

#### 3.2.4. *Vibrio parahaemolyticus*

**Figure 5:**
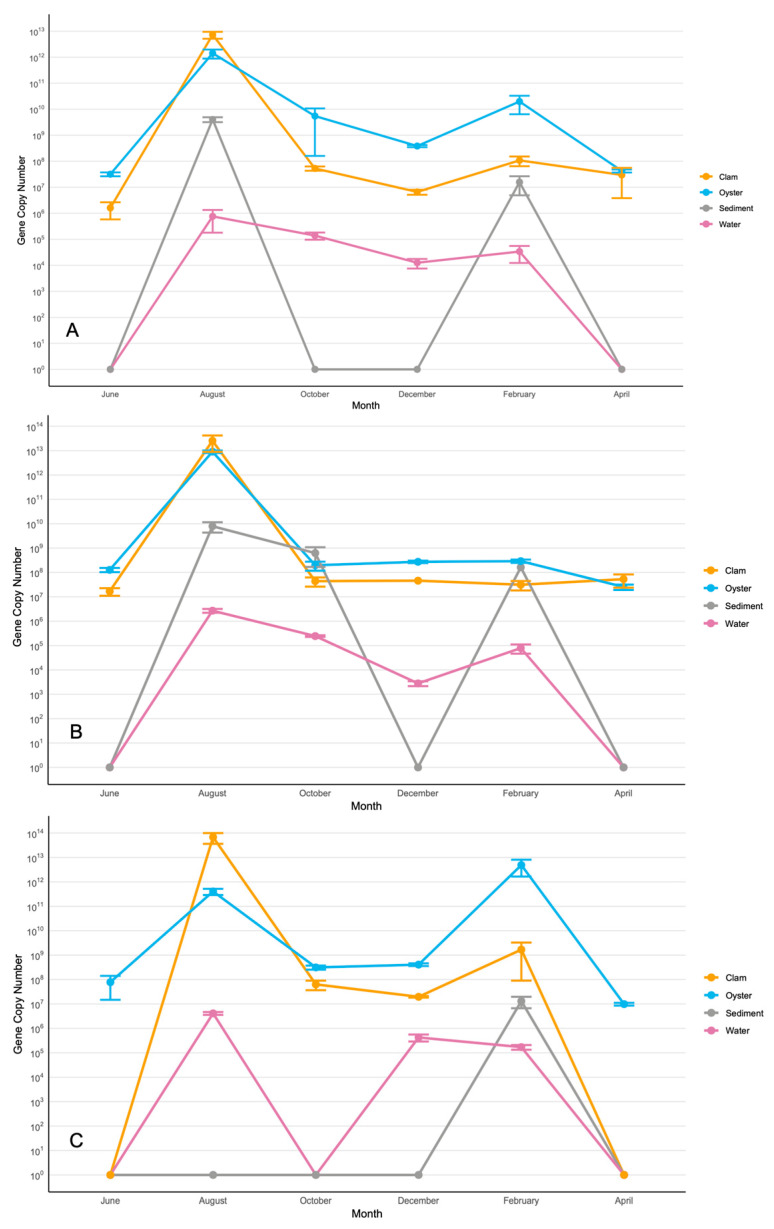
Copy numbers/g/mL of *V. parahaemolyticus* in Sites 1 (**A**), 2 (**B**), and 3 (**C**). The data for the figure were obtained from samples collected in triplicate from three segments of each site. Nine samples were collected at each site in each sampling month. Each triplicate was taken ~25 m apart to ensure an accurate representation of the site. Average copy numbers were displayed per 1 g of sediment, clams, and oysters and 1 mL of water. Error bars represent standard deviations (N = 9).

*V. parahaemolyticus* was the most prominent Harveyi-clade species in all the sites, with the highest abundance and most predictable spatial and temporal dynamics. This species was present in all environmental niches year-round. The spatial and temporal dynamics were remarkably similar at all the sites. The peaks of *V. parahaemolyticus* concentration simultaneously occurred in the water column, sediments, and bivalves in August and February (Figure 5). The highest concentration of *V. parahaemolyticus* in the water column also occurred in August at ~10^6^–10^7^ gene copies/mL (Figure 5).

In contrast to popular belief, *V. parahaemolyticus* was consistently present in mollusks, even in winter months. Unlike *V. splendidus*, this pathogen did not move out of the hosts and into sediments during the winter months. The highest concentrations of *V. parahaemolyticus* in clams and oysters were reached in August, at ~10^14^ gene copies/g and ~10^13^ gene copies/g, respectively (Figure 5B,C). The sediment concentration was the second highest, reaching ~10^10^ gene copies/g in August (Figure 5A,B).

### 3.3. Impact of Water Parameters on the Distribution and Abundance of Targeted Vibrio Species

In the water column, temperature, water acidity, and oxygen concentration uniformly impacted all the *Vibrio* species (Table 3), making those reliable predictors for the abundance of Harveyi-clade and *V. splendidus* pathogens. As expected, all the species positively reacted to the increase in temperature. Observed reverse correlations of all the species to pH and oxygen were not expected. *V. harveyi*/*V. campbellii* were the only species with a reversed correlation to salinity, conductivity, TDS, and potential water density, likely indicating the species’ preference for a less saline environment. Due to a single detection of *V. alginolyticus* in the water column and only at Site 3, no correlation between the environmental parameters and its concentration in the water column could be established.

In the sediments, all the species showed a negative correlation to dissolved oxygen (Table 4). The impacts of other water parameters on the abundance of *Vibrio* pathogens were species-specific, which separated *V. splendidus* from the Harveyi-clade species. *V. splendidus* demonstrated a strong positive correlation to pH and a moderate to strong negative correlation to all the other parameters. This was likely related to its seasonal cycles in which *V. splendidus* stays in the water table and bivalves for summer and spring, and moves to sediments in winter. Due to a lack of detection of *V. alginolyticus* in sediments, no correlation for this species could be established.

All the species associated with bivalves uniformly correlated to water parameters. An increase in temperature and salinity, and a decrease in pH and oxygen concentration corresponded to the abundance of Harveyi-clade species and *V. splendidus* in oysters (Table 5) and clams (Table 6). Therefore, the above parameters predict the abundance of the targeted species in the water column and bivalves, but not in sediments.

### 3.4. The Harveyi-Clade Carriers of Virulence Genes

The abundance of the *Vibrio* species was compared to the abundance of previously detected Harveyi-clade-specific virulence genes [5]. Concentrations of six virulent genes, *toxR*, *luxR*, *srp*, *vhh*, *vhhA* and *vhp*, were related to the concentrations of the Harveyi-clade species. The most consistent correlations were observed between the concentrations of *V.harveyi*/*V. campbellii* and *V. parahaemolyticus* to *luxR*, *vhhA,* and *vhp* genes (Table 7). The highest correspondence was between the abundance of these genes in water and sediments and the abundance of *V.harveyi*/*V. campbellii*. The second highest was observed between these genes and *V. parahaemolyticus*. These correlations were high, reliable, and site-specific, possibly indicating uneven distribution of potentially highly virulent strains of each species. In the water column of site 3 and to some extent site 1, but not in any other site or sediments, the abundance of *V. splendidus* also corresponded to the abundance of the virulent genes. While it was possible that *V. splendidus* also possessed virulence genes with sequences similar to those carried by the Harveyi clade, it was more likely that this correspondence was due to the abundance of *V. harveyi*/*V. campbellii* and *V. parahaemolyticus* in these two locations, and a coincidental presence of *V. splendidus* at these locations.

## 4. Discussion

### 4.1. Shared Patterns of the Targeted Vibrio Species

This study sought to assess the temporal and spatial distribution and abundance of Harveyi- and Splendidus-clade species in coastal Southeastern USA Atlantic waters. The species abundance was derived from the concentrations of species-specific genes. Each site was sampled at three locations in triplicate with nine samples collected from each site at each sampling event. This approach provides high credibility for the presented data. There was a remarkable similarity in the presence, distribution, and abundance of each species among the three sampling sites.

The highest frequency and abundance of each species targeted in this study was in summer. Several studies have shown a positive correlation between *Vibrio* concentrations and temperature [34,35,36]. Cantet et al. [37] monitored *Vibrio* spp. in a coastal region, with the highest detection in July and August, which were also the hottest months. The average copy numbers of species-specific genes were generally higher in the clams and oysters than in the water column and sediment. Oysters and clams are filter feeders; they can selectively or non-selectively accumulate microorganisms in their tissues. In an earlier study, selective bioaccumulation was observed for bacterial carriers of particular antibiotic-resistance genes in *Crassostrea virginica* oysters [38].

Water parameters predicted the presence and abundance of all the targeted species in the water column, but not in sediments. Temperature, water acidity, and oxygen concentration uniformly (negatively or positively) correlated to the presence of all targeted species in water, which makes those reliable predictors for the abundance of Harveyi-clade pathogens and *V. splendidus* in the water column. A positive correlation between the abundance of *Vibrio* pathogens and temperature has been reported in many studies and addressed above. A negative correlation with the pH has been reported in a handful of previous studies. The impact of pH on the growth of *V. parahaemolyticus* and *V. vulnificus* was recently described as nonlinear, with maximal growth rates at approximately pH 5.5, 7.0, and 8.2, in planktonic cultures and biofilms [39]. The authors also reported an interplay between pH and temperature. Barkovskii and Brown [5] demonstrated a negative correlation between the abundance of Harveyi-clade-specific virulence genes and pH.

In sediments, the correlation patterns were mixed, less pronounced, and appeared to be species-specific. Sediments were earlier reported as a haven for pathogens under stressful conditions [40]. Sediments may provide more favorable conditions for persistence and regrowth of pathogens via delivering protection from sunlight irradiation and predation, favorable nutrient conditions, and mitigating impacts of cardinal water parameters [41].

A reverse correlation between oxygen concentration and the abundance of Harveyi-clade pathogens and *V. splendidus* was observed in all the environmental matrices. The strongest negative correlation was observed in mollusks. Numerous studies reported that lower oxygen concentration promotes the synthesis of colonization factors and biofilm formation [42,43], likely causing the movement of *Vibrio* pathogens into bivalve hosts and their colonization. Overall, temperature, pH, and oxygen concentration consistently revealed positive or negative correlations to the abundance of Harveyi-clade pathogens and *V. splendidus* in oysters and clams. Therefore, they can serve as reliable indicators for predicting their abundance in bivalves.

Correlations between particular concentrations of virulence genes, *V.harveyi*/*V.campbelli* and *V. parahaemolyticus* were strong and statistically reliable but not in all the sites. A previous study found that infaunal burrows of an estuary in South Carolina are “hot spots” for pathogenic *V. parahaemolyticus* [44], whose presence was highly correlated with the virulence genes. It is possible that there were faunal burrows created by fiddler crabs in the sampling sites of this study that caused these “hot spots” in sediments for this and other carriers of virulence genes, but this does not explain the existence of “hot spots” in water samples. A random distribution of *tdh* and *trh* virulence genes and their *Vibrio* carriers between individual mollusks has also been previously observed [45].

### 4.2. V. splendidus

*V. splendidus* is a member of the Splendidus clade, is commonly found in Pacific coastal regions, and primarily infects bivalves, such as oysters and clams, causing mass mortality [14,26]. The usual hosts for this species are the Pacific oyster (*Crassostrea gigas*) and carpet shell clam (*Ruditapes decussatus*) [14,27]. There are no reports of this species in the Southeastern USA Atlantic, and for all the above reasons, it was included in this study.

*V. splendidus* was observed at a high frequency and concentration in the water column, *Crassostrea virginica* oysters, *Mercenaria mercenaria* clams, and sediments with an apparent seasonal trend. Similar correlations between its abundance in the two mollusk species and environmental parameters revealed a low, if any, host dependency of this species. In summer, its concentration in the mollusks was higher than that in the water column either because of selective or non-selective bioaccumulation [38] or because the mollusks provided a more hospitable environment for this pathogen in summer. In winter, no *V. splendidus* was detected in mollusks or water, but this species was abundant in sediments, suggesting sediments as the preferred habitat in winter.

Previous studies performed in the Adriatic have reported isolation of *V. splendidus* from water and hosts in winter [31,46]. The temperature might not be the only factor impacting the seasonal distribution of *V. splendidus*. In particular, the Adriatic and Southeastern USA Atlantic may have experienced different seasonal patterns in salinity, turbidity, pH, and conductivity that have essentially driven the migration of *V. splendidus* between the water column, mollusks, and sediments in our study. Observed strong positive correlation of *V. splendidus* to pH and a moderate to strong negative correlation to all the other parameters, likely related to its seasonal cycles in which *V. splendidus* stays in the water table and bivalves for summer and spring, and moves to sediments in winter. Sediments were earlier reported as a haven for pathogens under stressful conditions [40].

Only at one site and only in the water column did the concentration of *V. splendidus* correspond to the concentration of the major virulence genes. This suggests a coincidental event rather than inferring this species as a carrier of those genes.

### 4.3. V. alginolyticus

This species can grow under a broad range of salinity and temperature conditions [47]. This pathogen is a major contributor to mass mortality in *Crassostrea gigas* aquaculture [14,15] and fish species [16] in the Pacific. It is also known as a human pathogen, causing soft tissue and bloodstream infections [17]. This species was previously reported on the Atlantic USA coast [47], but we did not find reports on its environmental behavior there.

The observed peak of its concentration in oysters and clams in August was expected. *V. alginolyticus* is closely related to *V. parahaemolyticus* and mainly occurs in mollusks in the summer months, correlating with the temperature [48]. As *V. splendidus*, *V. alginolyticus* was detected at similarly high concentrations in oysters and clams in this study, demonstrating low to no host specificity. This species was detected in the water column at all sites, but only in April. This species was not detected in sediments. Due to these facts, it was impossible to establish a correlation between the virulence genes in these environments and the abundance of this species.

*V. alginolyticus* left bivalves in October but was absent from the water column until April. Neither did it move to the sediment like *V. splendidus* did. A possible explanation could be that *V. alginolyticus* moved to other hosts not researched in this study. Numerous articles show that various fish species are hosts for this pathogen [49,50]. Kataržytė et al. [51] reported red algae wrack as a hospitable environment for *V. alginolyticus*, and there is always a possibility that *V. alginolyticus* concentrations in sediments fell below the detection limit.

### 4.4. Vibrio harveyi/Vibrio campbellii

Among the Harveyi clade, *V. harveyi* and *V. campbellii* are the culprits for most of the outbreaks in aquaculture that are notorious for affecting shrimp and fish in Southeast Asia [52], leading to significant economic loss. Most infected species exhibit lethargy, luminescence, and tissue necrosis [22]. This disease has been shown to exhibit high mortality rates, which kills 80% of the infected population [22]. These species have also been found in mollusks, leading to rare incidences of vibriosis in humans [23]. *V. harveyi* and *V. campbellii* have been shown to share more than 99% similarity in 16S rRNA genes, indicating a recent common ancestor [53], and making it problematic to detect these species separately in the environment.

*V. harveyi* and *V. campbellii* have not been previously reported in coastal waters of the USA Atlantic. Despite its low concentration in sediments and, especially in the water column, the presence of these species revealed the highest correlation to the presence of the clade-specific genes. *V. harveyi* and *V. campbellii* share the same virulence factors [19]. Virulence genes such as *luxR*, *chiA*, *vhhA*, *toxR*, and *srp* were identified in these pathogens and *V. parahaemolyticus* [54], and those were the genes whose concentrations corresponded to the presence of these species in sediments and water, making them the most suspicious pathogens in our study. Most consistent correlations were observed between the concentrations of these pathogens and *luxR*, *vhhA,* and *vhp* genes. The *luxR* encodes for quorum sensing regulator and was reported in *V. harveyi, V. campbellii*, and *V. parahaemolyticus*, and related to these *Vibrio* species [55]. The *vhhA* encodes for hemolysin in Harveyi-clade species [56], and *vhp* is the metalloprotease gene of the Harveyi clade [6].

The fact that these species were not detected in oysters and clams may indicate that they have infected other species, e.g., shrimps, that are their preferred hosts [22], which were not included in this study. On the other hand, PCR amplification might have failed due to the presence of inhibitors in DNA extracted from the tissues of mollusks.

### 4.5. Vibrio parahaemolyticus

*V. parahaemolyticus* is the most well-known species among the clade members. This is primarily associated with gastroenteritis through the consumption of raw or undercooked seafood [57]. *V. parahaemolyticus* is the only clade member monitored by most of the States in oysters.

This pathogen was the most persistent in our study, being detected in 94% of all samples, and it revealed a year-round presence in all the environmental matrices. In an earlier study, Prescott and Barkovskii [29] consistently observed *V. parahaemolyticus* in water, sediments, and oysters collected in the same area. This and other studies [58] reflected similar seasonality in *V. parahaemolyticus* abundance. In this study, *V. parahaemolyticus* presented at high concentrations in oysters and clams, making an old saying about their food safety in months having an R in their names obsolete.

*V. parahaemolyticus* revealed the second strongest correlation, after *V. harveyi*/*V. campbellii*, with the concentrations of the virulence genes. This correlation was patchy as in the case of *V. harveyi*/*V. campbellii*. As for *V. harveyi*/*V. campbellii*, the most consistent positive correlations were observed between the concentrations of *V. parahaemolyticus luxR, vvhA*, and *vhp* virulence genes. Earlier, *luxR, vvhA*, and *vhp* were reported as shared between those three species [19,54,59]. The uneven correlations of their carriers between the sites and environmental niches suggested an uneven distribution of pathogenic strains within the species. Uneven distribution of pathogenic strains in sediments and bivalves has been previously reported [44,45].

This study revealed the seasonal dynamics of three Harveyi-clade species, *V. alginolyticus*, *V. parahaemolyticus*, *V. harveyi*/*V. campbellii*, and *V. splendidus* in the Southeastern USA Atlantic. The species were detected in water, bivalves (except for *V. harveyi*/*V. campbellii*), and sediments (except for *V. alginolyticus*). Although the highest species abundance was observed mostly in summer, the spatial dynamics varied between the species. This is the first report on *V. harveyi*/*V. campbellii* in Atlantic USA waters. *V. harveyi*/*V. campbellii* and *V. parahaemolyticus* were recognized as the most likely carriers of the Harveyi-clade-specific virulence genes previously detected in the study area. In the water column and bivalves, temperature, acidity, and oxygen concentration consistently (positively or negatively) correlated with the concentration of the species-specific to Harveyi-clade and *V. splendidus* genes and were suggested as reliable predictors for the abundance of these species. Except for the reverse correlation to oxygen concentration, the impact of other water parameters on the abundance of Harveyi-clade species and *V. splendidus* in sediments was species-specific, suggesting a lack of uniform environmental drivers controlling the movement of these species between the water column and sediments.

## 5. Conclusions

This study prioritizes screening of bivalves for *V. parahaemolyticus* due to its highest detection rates and demonstrated association with the clade-specific virulence genes. We suggest integrating real-time sensors (T, pH, O_2_) with periodic qPCR for species markers and virulence genes into the State monitoring program and in farmers’ practice. A solid applied study that operationalizes the relationship between water parameters and Vibrio risk in bivalve production areas, while also investigating a novel biogeographic contribution (*V. harveyi*/*V. campbellii* in the US Atlantic), is appropriate. That study could be used as a basis for predictive monitoring systems built on qPCR analysis and environmental sensors. Recommendations for aquaculture and public health practitioners could be developed from the proposed study.

## Figures and Tables

**Table 1:** Occurrence of *Vibrio* species in sediment and water in Sites 1–3.

Site/Targeted Species	Va	Vs	Vp	Vc & Vh
**Site 1**				
June Sediment	−	−	−	+
August Sediment	−	−	+	+
October Sediment	−	−	−	−
December Sediment	−	−	−	−
February Sediment	−	+	+	−
April Sediment	−	−	−	N/A
June Water	−	−	−	−
August Water	−	+	+	−
October Water	−	+	+	−
December Water	−	+	+	−
February Water	−	−	+	−
April Water	+	+	−	N/A
**Site 2**				
June Sediment	−	−	−	+
August Sediment	−	−	+	+
October Sediment	−	−	+	−
December Sediment	−	−	−	−
February Sediment	−	+	+	−
April Sediment	−	−	−	N/A
June Water	−	−	−	−
August Water	−	+	+	−
October Water	−	+	+	−
December Water	−	+	+	−
February Water	−	−	+	−
April Water	+	+	−	N/A
**Site 3**				
June Sediment	−	−	−	+
August Sediment	−	−	+	+
October Sediment	−	−	+	−
December Sediment	−	−	−	−
February Sediment	−	+	+	−
April Sediment	−	−	−	N/A
June Water	−	−	−	+
August Water	−	+	+	−
October Water	−	+	−	−
December Water	−	+	+	−
February Water	−	−	+	−
April Water	+	+	−	N/A
**Detection Frequency**	3/36 (8.33%)	15/36 (41.67%)	19/36 (52.78%)	7/36 (19.44%)
**Sediment Total**	0/18 (0%)	3/18 (16.67%)	8/18 (44.44%)	6/18 (33.33%)
**Water Total**	3/18 (16.67%)	12/18 (66.67%)	11/18 (61.11%)	1/18 (5.55%)

Captions: Va: *V. alginolyticus*, Vs: *V. splendidus*, Vp: *V. parahaemolyticus*, Vc & Vh: *V. campbellii* and *V. harveyi*. N/A: not analyzed.

**Table 2:** Occurrence of *Vibrio* species in clams and oysters in Sites 1–3.

Site/Targeted Species	Va	Vs	Vp
**Site 1**			
June Clam	−	−	+
August Clam	+	+	+
October Clam	−	−	+
December Clam	−	−	+
February Clam	−	−	+
April Clam	−	−	+
June Oyster	−	−	+
August Oyster	+	+	+
October Oyster	−	−	+
December Oyster	−	−	+
February Oyster	−	−	+
April Oyster	−	−	+
**Site 2**			
June Clam	−	−	+
August Clam	+	+	+
October Clam	−	−	+
December Clam	−	−	+
February Clam	−	−	+
April Clam	+	−	+
June Oyster	−	−	+
August Oyster	+	+	+
October Oyster	−	−	+
December Oyster	−	−	+
February Oyster	−	−	+
April Oyster	−	−	+
**Site 3**			
June Clam	−	−	−
August Clam	+	+	+
October Clam	−	−	+
December Clam	−	−	+
February Clam	−	−	+
April Clam	−	−	−
June Oyster	−	−	+
August Oyster	+	+	+
October Oyster	−	−	+
December Oyster	−	−	+
February Oyster	−	−	+
April Oyster	−	−	+
**Detection Frequency**	7/36 (19.44%)	6/36 (16.67%)	34/36 (94.44%)
**Clam Total**	4/18 (22.22%)	3/18 (16.67%)	16/18 (88.89%)
**Oyster Total**	3/18 (16.67%)	3/18 (16.67%)	18/18 (100%)

Captions: Va: *V. alginolyticus*, Vs: *V. splendidus*, Vp: *V. parahaemolyticus*. No *V. campbellii* and *V. harveyi* were ever detected.

**Table 3:** Correlation (Pearson’s Coefficient) between water parameters and abundance of *Vibrio* species in the water column.

Pearson Coeff.	pH	Temp. (°C)	Salinity (ppt)	Turbidity (NTU)	dOxygen (mg/L)	Conduct (mS/cm)	TDS (g/L)	Water Density (g/cm^3^)
Vp	−0.425	0.597 *	0.331	−0.210	−0.375	0.256	0.259	−0.127
Va	0.000	0.000	0.000	0.000	0.000	0.000	0.000	0.000
Vs	−0.351	0.408	0.455	−0.070	−0.182	0.434	0.386	0.070
Vc & Vh	−0.745 **	0.462	−0.325	0.456	−0.485	−0.372	−0.430	−0.598 *

Captions: Orange, a moderate positive correlation; Dark Blue: a strong negative correlation; Light Blue: a modest negative correlation; No Color, no correlation. * *p*-value of 0.05, ** *p*-value of 0.01, no asterisk indicates no significance of *p*-values. Va: *V. alginolyticus*, Vs: *V. splendidus*, Vp: *V. parahaemolyticus*, and Vc & Vh: *V. campbellii* and *V. harveyi*.

**Table 4:** Correlation (Pearson’s Coefficient) between water parameters and abundance of *Vibrio* species in sediment.

Pearson Coefficient	pH	Temp (°C)	Salinity (ppt)	Turbidity (NTU)	Dissolved Oxygen (mg/L)	Conduct (mS/cm)	TDS (g/L)	Water Density (g/cm^3^)
Vp	−0.455	0.636 *	0.315	−0.215	−0.409	0.227	0.240	−0.166
Va	0.000	0.000	0.000	0.000	0.000	0.000	0.000	0.000
Vs	0.602 *	−0.134	−0.816 ***	−0.867 ***	−0.305	−0.736 **	−0.738 **	−0.365
Vc & Vh	−0.724 **	0.437	−0.334	0.460	−0.467	−0.378	−0.435	−0.588 *

Captions: Va: *V. alginolyticus*, Vs: *V. splendidus*, and Vp: *V. parahaemolyticus.* Orange: a moderate positive correlation; Dark Blue: a strong negative correlation; Light Blue: a modest negative correlation; No Color, no correlation. * *p*-value of 0.05, ** *p*-value of 0.01, *** *p*-value of 0.001, no asterisk indicates no significance of *p*-values.

**Table 5:** Correlation (Pearson’s Coefficient) between water parameters and abundance of *Vibrio* species in oysters.

Pearson Coefficient	pH	Temp (°C)	Salinity (ppt)	Turbidity (NTU)	dOxygen (mg/L)	Conduct (mS/cm)	TDS (g/L)	Water Density (g/cm^3^)
Vp	−0.196	0.579 *	−0.064	−0.601 *	−0.559 *	−0.108	−0.107	−0.339
Va	−0.463	0.632 *	0.303	−0.205	−0.415	0.224	0.225	−0.171
Vs	−0.464	0.632 *	0.303	−0.205	−0.416	0.224	0.225	−0.172

Captions: Va: *V. alginolyticus*, Vs: *V. splendidus*, and Vp: *V. parahaemolyticus*. Light Orange: a modest positive correlation; Light Blue: a modest negative correlation; No Color, no correlation. * *p*-value of 0.05, no asterisk indicates no significance of *p*-values.

**Table 6:** Correlation (Pearson’s Coefficient) between water parameters and abundance of *Vibrio* species in clams.

Pearson Coefficient	pH	Temp (°C)	Salinity (ppt)	Turbidity (NTU)	dOxygen (mg/L)	Conduct (mS/cm)	TDS (g/L)	Water Density (g/cm^3^)
Vp	−0.464	0.632 *	0.303	−0.205	−0.415	0.224	0.225	−0.171
Va	−0.464	0.632 *	0.303	−0.205	−0.415	0.224	0.225	−0.171
Vs	−0.464	0.632 *	0.303	−0.205	−0.415	0.224	0.225	−0.171

Captions: Va: *V. alginolyticus*, Vs: *V. splendidus*, and Vp: *V. parahaemolyticuss.* Orange: a moderate positive correlation; Dark Blue: a strong negative correlation; Light Blue: a modest negative correlation; No Color, no correlation. * *p*-value of 0.05, and No asterisk indicates no significance of *p*-values.

**Table 7:** Correlations (Pearson’s Coefficient) between copy numbers of the virulence genes and species-specific copy numbers.

Pearson Coefficient Water	Site 1—Water	Site 2—Water	Site 3—Water	Site 1—Sediment	Site 2—Sediment	Site 3—Sediment
Va ToxR	NA	NA	NA	NA	NA	NA
Va LuxR	NA	NA	NA	NA	NA	NA
Va Srp	NA	NA	NA	NA	NA	NA
Va vhhA	NA	NA	NA	NA	NA	NA
Va Vhp	NA	NA	NA	NA	NA	NA
Va vhh	NA	NA	NA	NA	NA	NA
Vs toxR	−0.266628	−0.178348	0.614342 *	−0.369999	−0.308843	−0.237613
Vs luxR	0.576408 *	−0.309255	0.956181 ***	−0.300033	−0.243797	−0.379674
Vs srp	−0.267089	−0.276635	0.911818 ***	−0.314119	−0.029936	−0.251624
Vs vhhA	0.636287 *	0.336443	0.956532 ***	−0.253705	−0.418526	−0.255721
Vs vhp	0.914971 ***	−0.288870	0.956312 ***	−0.305423	−0.359860	−0.244952
Vs vhh	−0.182311	0.029843	0.940769 ***	−0.237935	−0.099112	−0.315662
Vp toxR	−0.326406	−0.198623	0.675464 **	−0.376410	0.993103 ***	−0.237613
Vp luxR	0.517126 *	−0.329730	0.994786 ***	0.985776 ***	0.997616 ***	−0.379674
Vp srp	−0.326671	−0.298497	0.961058 ***	−0.031231	0.962990 ***	−0.251624
Vp vhhA	0.577854 *	0.319396	0.994962 ***	−0.245787	0.821006 ***	−0.255721
Vp vhp	0.908499 ***	−0.309028	0.994787 ***	−0.232470	0.042736	−0.244952
Vp vhh	−0.238271	0.003321	0.985684 ***	−0.215360	0.524304 *	−0.315662
Vh/Vc toxR	−0.250000	0.991771 ***	0.564862 *	0.864884 ***	0.798366 ***	−0.252737
Vh/Vc luxR	0.590599 *	0.999925 ***	−0.171827	−0.004137	0.839814 ***	0.863118 ***
Vh/Vc srp	−0.250494	0.998538 ***	−0.198096	0.989971 ***	0.820817 ***	−0.241597
Vh/Vc vhhA	0.649705 **	0.790431 ***	−0.171463	0.997152 ***	0.998322 ***	−0.234020
Vh/Vc vhp	0.915609 ***	0.999913 ***	−0.172967	0.983395 ***	−0.072447	−0.259005
Vh/Vc vhh	−0.165745	0.565125 *	−0.097561	0.998677 ***	−0.031420	−0.336497

Captions: Va: *V. alginolyticus*, Vs: *V. splendidus*, Vp: *V. parahaemolyticus*, and Vc & Vh: *V. campbellii* and *V. harveyi*. Dark Orange: a strong positive correlation, Light Orange: a modest positive correlation, Light Blue: a modest negative correlation. * *p*-value > 0.05, ** *p*-value ≤ 0.01, and *** *p*-value ≤ 0.001. NA: not applicable.

## Data Availability

The original contributions presented in this study are included in the article/Supplementary Materials. Further inquiries can be directed to the corresponding author.

## Supplementary Materials

The following supporting information can be downloaded at: https://www.mdpi.com/article/10.3390/microorganisms13092167/s1, Table S1: Water parameters recorded in the study; Table S2: Sequences and amplicons of the species-specific primers utilized in this study; Table S3: PCR and qPCR conditions for species-specific primers utilized in this study.
