# Supplementary material for: Water Parameters Predicting the Seasonal and Spatial Dynamics of the Vibrio Harveyi- and Splendidus-Clade Pathogens
Source: Microorganisms. 2025 Sep 17;13(9):2167. doi: 10.3390/microorganisms13092167 (PMC12472194; doi:10.3390/microorganisms13092167)
Supplement: Supplementary file 1 [file microorganisms-13-02167-s001.zip › microorganisms-3790542-supplementary.pdf]

Supplemental

Table S1. Water parameters recorded in the study

Water Parameters	pH	Temperature (Celsius)	Salinity (ppt)	Turbidity (NTU)	Dissolved Oxygen (mg/L)	Conductivity (mS/cm)	TDS (g/L)	Potential Water Desnity
**June**								
Site 1	7.1	27.63	18.3	23.6	1.24	31.50	18.1	10.2
Site 2	7.23	26.33	22.4	21.1	1.18	35.70	22.9	11.4
Site 3	7.00	28.05	23.5	22.5	1.26	38.30	23.4	13.6
**Average**	**7.11**	**27.34**	**21.40**	**22.40**	**1.23**	**35.17**	**21.47**	**11.73**
**August**								
Site 1	7.11	29.42	23.8	15.7	1.5	38.8	24.4	13.8
Site 2	7.10	31.05	24.2	18.4	1.43	39.4	25.1	13.9
Site 3	7.30	30.8	24.9	17.3	0.87	40.0	24.4	14.1
**Average**	**7.17**	**30.42**	**24.30**	**17.13**	**1.27**	**39.40**	**24.63**	**13.93**
**October**								
Site 1	7.28	20.65	24.3	18.9	2.54	39.5	25.3	15.8
Site 2	7.33	20.95	24.6	20.2	2.30	39.6	25.6	17.3
Site 3	7.37	21.85	25.1	20.3	2.90	39.0	26.1	16.2
**Average**	**7.33**	**21.15**	**24.67**	**19.8**	**2.58**	**39.37**	**25.67**	**16.43**
**December**								
Site 1	7.43	11.53	23.8	20.4	3.6	43.8	26.7	20.2
Site 2	7.43	11.52	26.6	25.2	3.51	43.5	26.9	20.0
Site 3	7.36	11.32	24.8	21.7	3.22	40.4	24.4	18.1
**Average**	**7.41**	**11.46**	**25.07**	**22.43**	**3.44**	**42.57**	**26.00**	**19.43**
**February**								
Site 1	7.52	21.29	19.5	17.6	1.5	33.0	20.1	12.8
Site 2	7.37	20.38	21.4	11.2	0.9	35.6	21.7	14.5
Site 3	7.47	19.54	17.0	7.0	1.98	29.6	18.4	11.3
**Average**	**7.45**	**20.40**	**19.30**	**11.93**	**1.46**	**32.73**	**20.07**	**12.87**

Table S2. Sequences and amplicons of the species-specific primers utilized in this study

Target Species	Primer Gene/Protein	Primer Sequence	Amplicon Size	Reference
V. splendidus	*recA*	F: TGARAARCARTTYGTTAAAGG<br>R: TCRCCNTTRTAGCTRTACC	837	Torresi et al. (2011)
V. parahaemolyticus	*tlh*	F: ACTCAACACAAGAAGAGATCGACAA<br>R: GATGAGCGGTTGATGTCCAA	208	Nordstrom et al. (2007)
V. alginolyticus	VA 1198230 whole genome shotgun sequence	F: ACGGC ATTGG AAATT GCGAC TG<br>R: TACCC GTCTC ACGAG CCCAA G	199	Kim et al. (2015)
V. harveyi & V. campbellii	*rpoA*	F: CGTAGCTGAAGGCAAAGATGA<br>R: AAGCTGGAACATAACCACGA	197	Ruwandeepika et al. (2011)

Table S3. PCR and qPCR conditions for species-specific primers utilized in this study.

**Species**	**Primer**	**Activation**	**Denaturation**	**Annealing**	**Elongation**	**Extension**	**Cycles**	**References**
Vs	*recA*	10˚C 10 m	94˚C f 1 m	53˚C 90 s	72˚C 150 s	72˚C 10 m	35	Torresi et al. (2011)
Vp	*tlh*	-	95˚C for 1 m	95˚C 5 s	59˚C 45 s	72˚C 5 m	40	Nordstrom et al. (2007)
Va	VA 1198230	94˚C 5 m	94°C for 30 s	60˚C 30 s	72˚C 30 s	-	25	Kim et al. (2015)
Vh/Vc	*rpoA*	50˚C 2 m	95˚C 10 m<br>95˚C 15 s	55˚C 20 s	72˚C 30 s	-	45	Ruwandeepika et al. (2011)

Captions: Va: *V. alginolyticus*, Vs: *V. splendidus*, Vp: *V. parahaemolyticus,* Vc/Vh: *V. campbellii* and *V. harveyi*
